# Non-contrast-enhanced silent magnetic resonance angiography for assessing cerebral aneurysms after PulseRider treatment

**DOI:** 10.1007/s11604-022-01276-z

**Published:** 2022-04-17

**Authors:** Tomoaki Suzuki, Hitoshi Hasegawa, Kazuhiro Ando, Kohei Shibuya, Haruhiko Takahashi, Shoji Saito, Makoto Oishi, Yukihiko Fujii

**Affiliations:** Department of Neurosurgery, Brain Research Institute, Niigata University, 1-757 Asahimachi-Dori, Niigata, 951-8585 Japan

**Keywords:** PulseRider, UTE-MRA, Silent MRA, Metal artifact, Cerebral aneurysm

## Abstract

**Purpose:**

Conventional time-of-flight (TOF) magnetic resonance angiography (MRA) failed to depict clear visualization of coiled cerebral aneurysms with PulseRider due to metal-induced susceptibility artifacts. Our aim was to overcome the metal artifact using a novel imaging technique of non-contrast-enhanced ultrashort echo-time magnetic resonance angiography (UTE-MRA).

**Materials and methods:**

Five unruptured intracranial aneurysms were treated using PulseRider and the patients underwent silent MRA (UTE-MRA). The images were compared with TOF-MRA and digital subtraction angiography (DSA).

**Results:**

Silent MRA can visualize the residual cavity of the coiled aneurysms, which was not well visualized and rather defective when using TOF-MRA. While a segment of the proximal marker composed of stainless steel was poorly visualized, the other parts of the parent artery and the arteries of bifurcation, including the aneurysmal neck, were clearly visualized, equivalent to that of DSA.

**Conclusions:**

UTE-MRA achieves better visualization of cerebral aneurysms after PulseRider treatment than TOF-MRA.

## Introduction

**Fig. 1:**
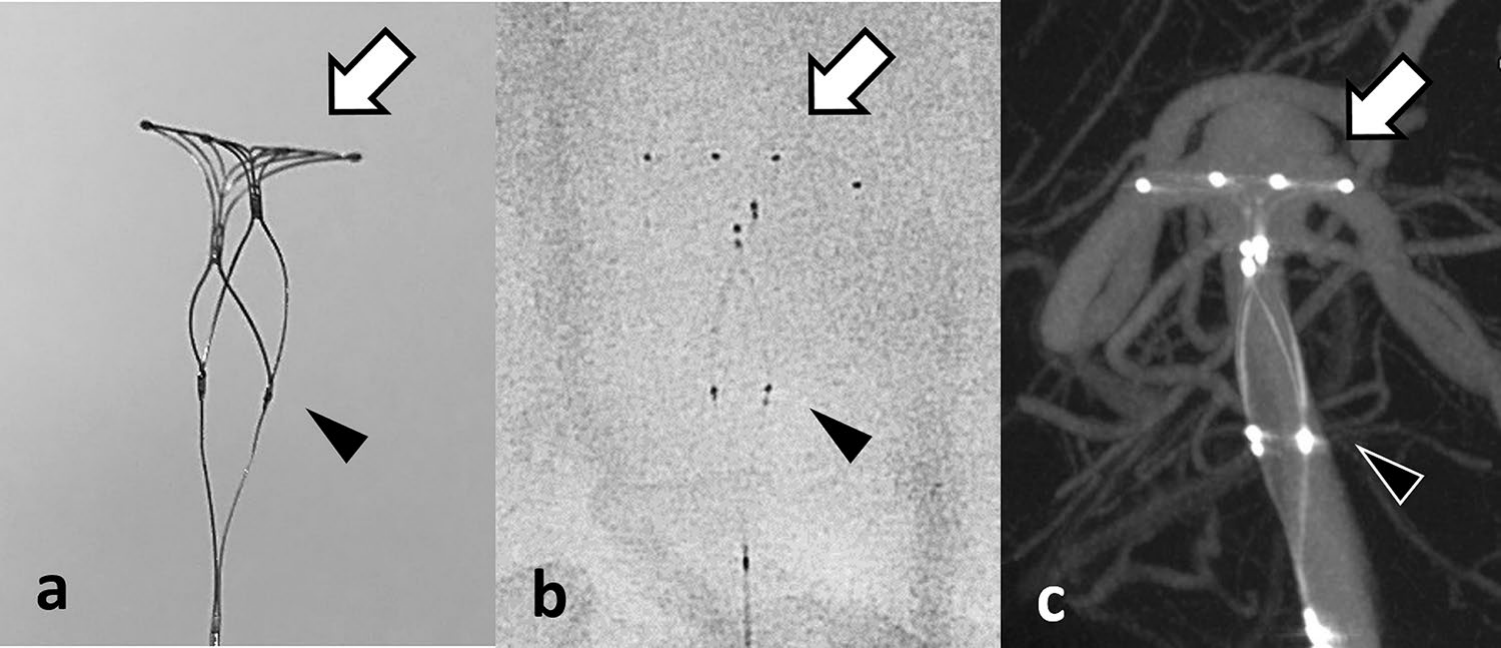
**a** Entire image of PulseRider (T-shaped implant). The arch (white arrow) provides the coverage of the aneurysm neck, which is made of nitinol with four platinum/iridium radiopaque markers. There are T and Y shapes with 8- and 10-mm wide arches, respectively. PulseRider has two anchor sizes labeled according to the parent vessel (below the branches): 2.7–3.5 mm and 3.5–4.5 mm. The proximal markers (black arrowhead) are attached with delivery wire and denote the detachment zone, made from stainless steel. **b** Native DSA image of PulseRider. **c** CBCT image of PulseRider in BA aneurysm. Four radiopaque markers (white arrow) are seen in the PulseRider arch, and two proximal markers (black arrowhead) are attached with delivery wire. *DSA* digital subtraction angiography, *CBCT* cone-beam computed tomography, *BA* basilar artery

The PulseRider Aneurysm Neck Reconstruction Device (PulseRider; Cerenovus, Irvine, CA, USA) is a novel endovascular device for wide-necked bifurcation aneurysms with known efficacy for use in various locations [1, 2]. The PulseRider is a self-expanding implant made of nitinol (nickel titanium) with a unique design whereby two petals formed by numerous struts span the aneurysm neck, and additional struts are oriented perpendicular to the neck-bridging portion of the device along with four overlapping saddle markers to support the proximal parent vessel lumen (Fig. 1) [3]. Most of the implanted device’s surface area covers the neck of the aneurysm, which must be cautiously monitored to prevent recanalization. Time-of-flight magnetic resonance angiography (TOF-MRA) is conventionally performed for non-invasive evaluation; however, metal artifacts make assessing the residual cavity after endovascular surgery difficult. PulseRider is mainly composed of nitinol and platinum/iridium, with up to 90% less metal than conventional stents. However, even with the metal reduction, it is difficult to clearly visualize flow signals at the neck of the aneurysm and the parent and bifurcation arteries [4, 5]. Recently, a newly developed non-contrast-enhanced ultra-short echo-time MRA (UTE-MRA) has been introduced and reportedly resolves metallic artifact-associated problems [6–8]. UTE-MRA has the potential to visualize residual cavities to a degree similar to or even superior to that of cerebral angiography. Previous reports have described the use of UTE-MRA in endovascular surgery; however, UTE-MRA for cerebral aneurysms coiled with PulseRider has not been reported. The aim of this study is to describe the successful visualization of coiled aneurysms with PulseRider using a novel UTE-MRA imaging technique.

## Materials and methods

### Patient and aneurysm characteristics

From January 2021 to August 2021, five consecutive patients were treated by coil embolization with PulseRider (three basilar arteries, one anterior communicating artery, and one middle cerebral artery). Table 1 shows patients’ characteristics, including aneurysm location, aneurysm size, and PulseRider size. TOF-MRA and silent MRA (UTE-MRA) were performed to evaluate the residual flow in the aneurysm sac and were compared with DSA images.

**Table 1:** Clinical information

Case	Age/sex	Location	Dome size (mm)	Neck size (mm)	D/N	Size of PulseRider (mm)
1	75/F	BA	6.2	6.6	0.9	10 T/3.5–4.5
2	52/M	BA	6.7	5.1	1.3	10 T/3.5–4.5
3	47/F	BA	7	5.6	1.3	8 T/2.7–3.5
4	66/M	Acom	9.5	5	1.9	10 T/2.7–3.5
5	73/M	MCA	5.3	4.5	1.2	8 T/2.7–3.5

*BA* basilar artery, *AcomA* anterior communicating artery, *MCA* middle cerebral artery

10 T: T shape with 10 mm-wide arch, 8 T: T shape with 8 mm-wide arch

PulseRider has two anchor sizes labeled with the intended parent vessel (below the branches) diameters 3.5–4.5 mm or 2.7–3.5 mm

### Imaging techniques

Silent MRA was first introduced as UTE-MRA with novel metal artifact reduction for intracranial aneurysms after endovascular surgery [6]. It was completed in a single scanning session using a 3-T magnetic resonance imaging (MRI) scanner and a UTE sequence with combined arterial spin labelling (ASL). The UTE sequence minimized phase dispersion and decreased the magnetic susceptibility of the coils and stents. ASL was used as a preparation pulse to visualize mild flow-signal changes. Control images were acquired before labeling the pulse. The control and labeled images were then subtracted to yield an angiographic image, improving the visualization of blood flow.

In our hospital, the scan parameters of silent MRA (Discovery MR 750w 3.0T; GE Healthcare, Waukesha, WI, USA) are as follows: repetition time (TR), 791 ms; echo time (TE), 0.020 ms; flip angle, 5°; field of view, 180 × 180 mm^2^; matrix, 150 × 150; section thickness, 1.2 mm; number of excitations, 1.0; bandwidth, 31.2 kHz; acquisition time: 5.5–6.5 min. The scan parameters of the 3D TOF-MRA were as follows: TR, 20 ms; TE, 3.4 ms, flip angle, 16°; field of view, 200 × 200 mm^2^; matrix, 384 × 224; section thickness, 1.0 mm; number of excitations, 0.85; bandwidth, 50.0 kHz; acquisition time: 4 min 40 s (3 slabs; overlap between slabs: 13 sections; 1 slab: 42 sections). DSA of catheter-based intra-arterial cerebral angiography was performed using the AXIOM Artis Zee BA Twin (Siemens Healthineers, Erlangen, Germany).

### Ethical approval

The study was approved by the institutional review board of our hospital and conducted in accordance with the principles of the Declaration of Helsinki. Written informed consent was waived due to the retrospective design of the study.

### Results: images of representative cases

**Fig. 2:**
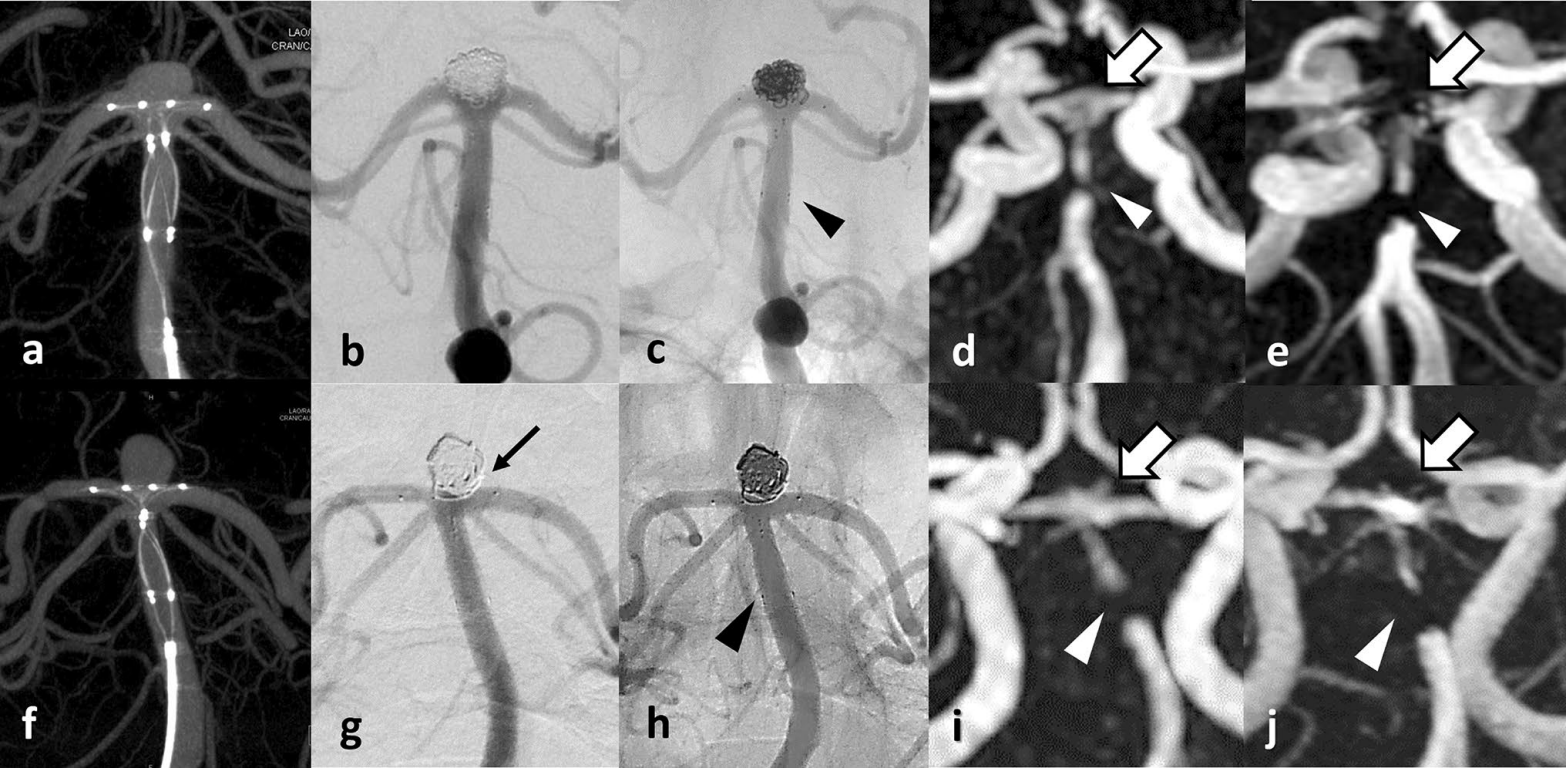
A 75-year-old female patient with an unruptured BA tip aneurysm (Case 1). **a** CBCT after PulseRider placement (T-shape, 10 mm, 3.5–4.5 mm). **b** Postoperative DSA image shows successful coil embolization with PulseRider. **c** Postoperative native DSA image. Black arrowhead indicates the proximal marker. **d** Silent MRA clearly shows the aneurysmal neck and parent artery at the bifurcation (white arrow). The segment of the proximal marker is poorly depicted (white arrowhead). **e** TOF-MRA failed to detect the area signal around the aneurysmal neck shown by the PulseRider device (white arrow). The segment of the proximal marker is poorly depicted (white arrowhead). A 52-year-old male patient with an unruptured BA tip aneurysm (Case 2). **f** CBCT after PulseRider placement (T shape, 10 mm, 3.5–4.5 mm). **g** Postoperative DSA image after coil embolization with PulseRider. The black arrow indicates a small neck remnant. **h** Postoperative native DSA image. Black arrowhead indicates the proximal marker. **i** Silent MRA clearly shows a small neck remnant and parent artery in the bifurcation (white arrow). The segment of the proximal marker is poorly depicted (white arrowhead). **j** TOF-MRA failed to detect the remnant neck clearly (white arrow). The segment of the proximal marker is poorly depicted (white arrowhead). *BA* basilar artery, *CBCT* cone-beam computed tomography, *DSA* digital subtraction angiography, *MRA* magnetic resonance angiography, *TOF* time-of-flight

**Fig. 3:**
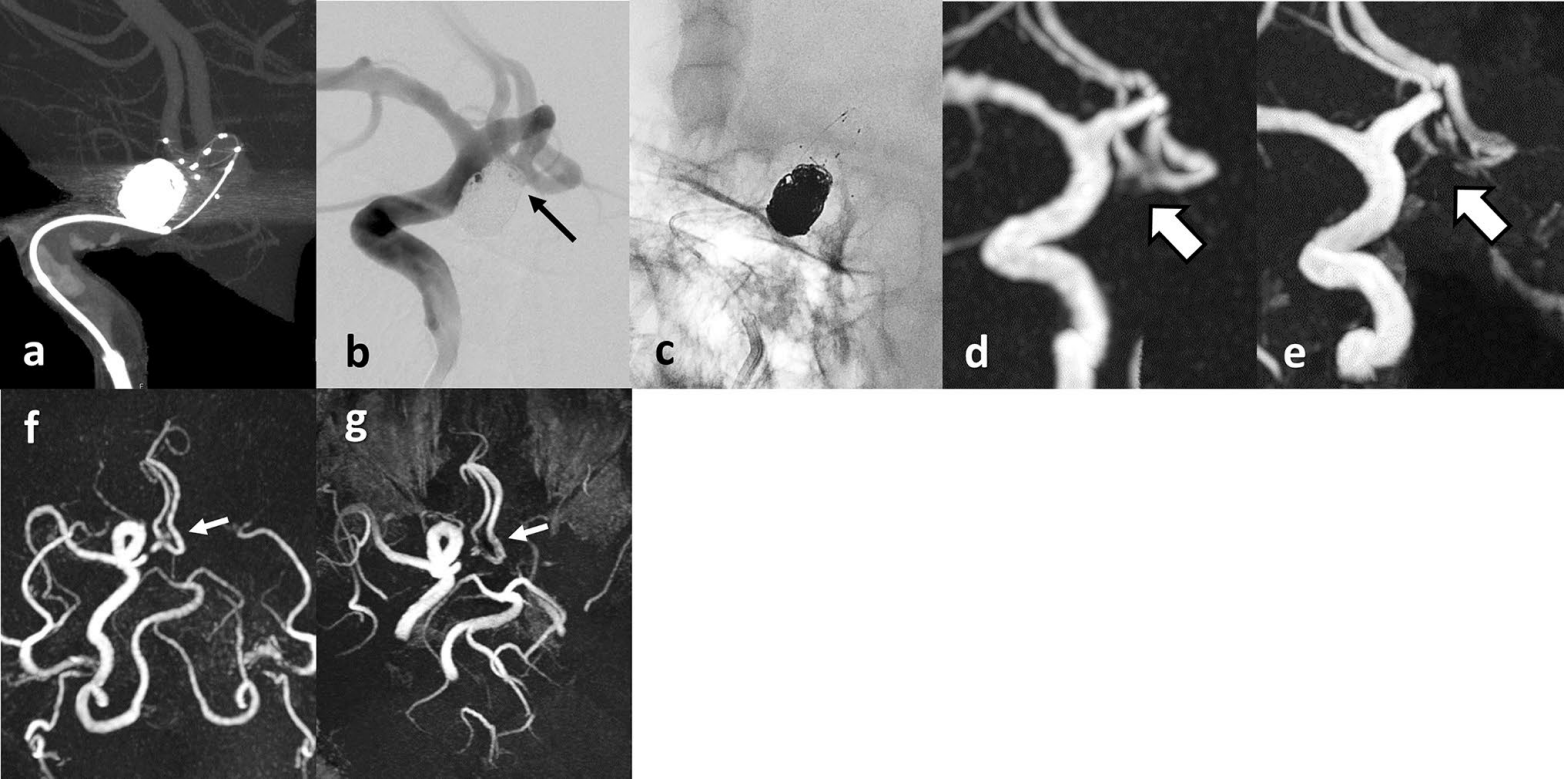
A 66-year-old male patient with a recurrent unruptured AcomA coiled aneurysm (Case 4). **a** CBCT after PulseRider placement (T shape, 10 mm, 2.7–3.5 mm). **b** Postoperative DSA image after coil embolization with PulseRider. The black arrow indicates a small neck remnant. **c** Postoperative native image after coil embolization with PulseRider. **d** Silent MRA image clearly shows a small neck remnant and parent artery in the bifurcation (white arrow). **e** TOF-MRA failed to depict the area signal shown by the PulseRider device (white arrow). **f** The entire image of silent MRA (axial view). A small neck remnant was visualized (white arrow). **g** The entire image of TOF-MRA (axial view). The signal around the aneurysmal neck was not detected (white arrow). *AcomA* anterior communicating artery, *CBCT* cone-beam computed tomography, *DSA* digital subtraction angiography, *MRA* magnetic resonance angiography, *TOF* time-of-flight

**Fig. 4:**
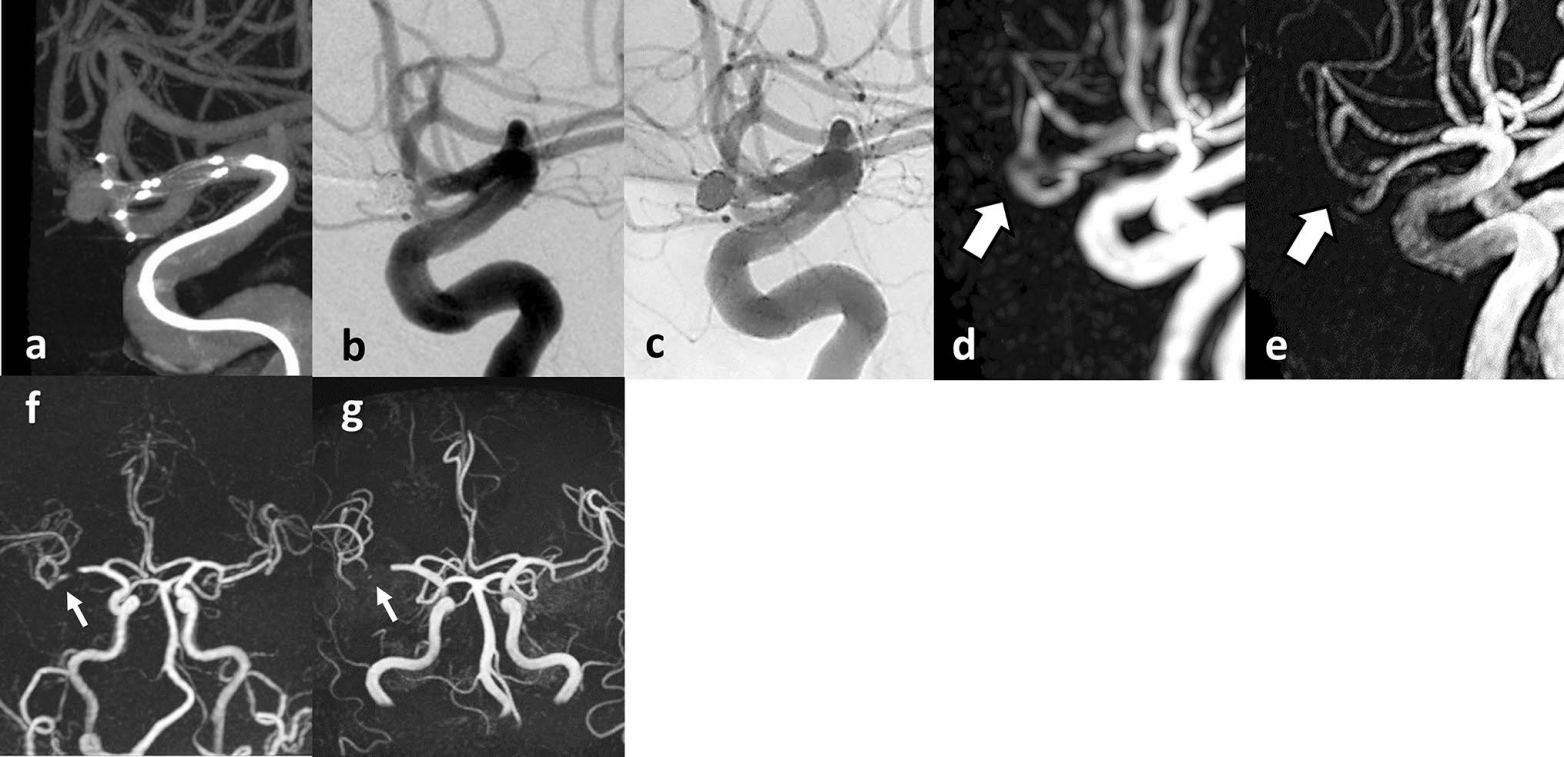
A 73-year-old male patient with an unruptured right MCA bifurcation aneurysm (Case 5). **a** CBCT after PulseRider placement (T shape, 8 mm, 2.7–3.5 mm). **b** Postoperative DSA image after coil embolization with PulseRider. **c** Postoperative native DSA image after coil embolization with PulseRider. **d** Silent MRA image shows the area signal at the aneurysmal neck and parent artery in the bifurcation (white arrow). **e** TOF-MRA failed to depict the area signal shown by the PulseRider device (white arrow). **f** The entire image of silent MRA. The signal around the aneurysmal neck was visualized (white arrow). **g** The entire image of TOF-MRA (anteroposterior view). TOF-MRA failed to demonstrate the total area signal at the PulseRider placement location (white arrow). *MCA* middle cerebral artery, *CBCT* cone-beam computed tomography, *DSA* digital subtraction angiography, *MRA* magnetic resonance angiography, *TOF* time-of-flight

**Fig. 5:**
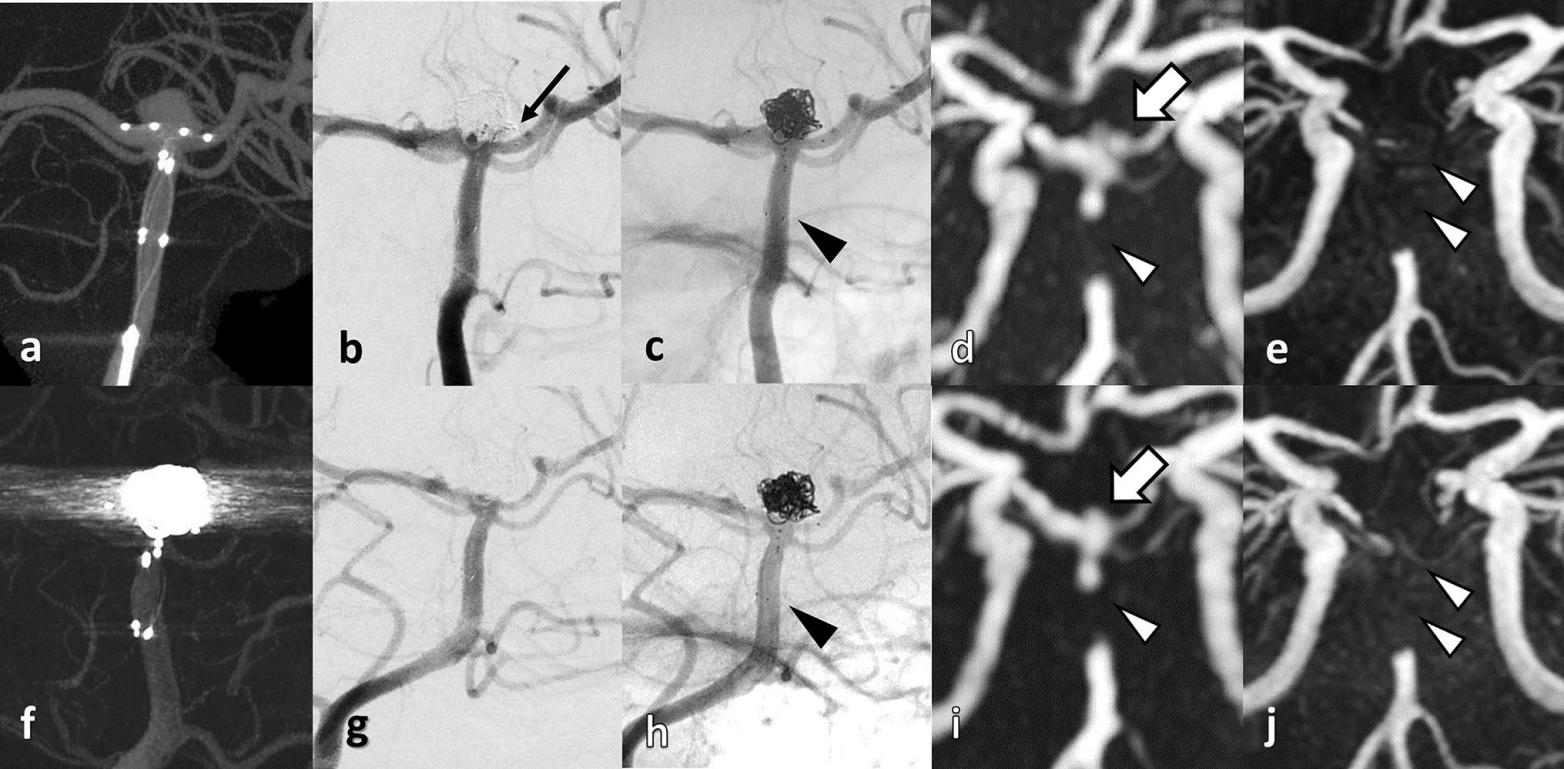
A 47-year-old female patient with an unruptured BA tip aneurysm (Case 3). **a** CBCT after PulseRider placement (T shape, 8 mm, 2.7–3.5 mm). **b** Postoperative DSA image after coil embolization with PulseRider. The black arrow indicates a small neck remnant. **c** Postoperative native DSA image. Black arrowhead indicates the proximal marker. **d** Silent MRA clearly shows a small neck remnant and parent artery in the bifurcation (white arrow). The segment of the proximal marker is poorly depicted (white arrowhead). **e** TOF-MRA failed to demonstrate the total area signal at the PulseRider placement location (double white arrowheads). Images at 6-month follow-up after PulseRider treatment. **f** CBCT of coiled aneurysm with PulseRider. **g** DSA image demonstrating complete occlusion of the aneurysm. **h** Native DSA image. Black arrowhead indicates the proximal marker. **i** Silent MRA image shows the occlusion of the residual neck similar to the DSA image (white arrow). The segment of the proximal marker is poorly depicted (white arrowhead). **j** TOF-MRA shows the same region with loss of signal as shown at the initial session (double white arrowheads). *BA* basilar artery, *CBCT* cone-beam computed tomography, *DSA* digital subtraction angiography, *MRA* magnetic resonance angiography, *TOF* time-of-flight

In all cases, PulseRider was successfully placed in the extra-aneurysmal position and coil embolization was performed with no intraprocedural complications. Silent MRA was able to adequately visualize the residual cavity of the coiled aneurysms similar to DSA, even in areas that were not properly visualized and defective when using TOF-MRA (Figs. 2, 3, 4, 5). A segment of the proximal marker was poorly visualized even when using silent MRA; however, other parts of the parent artery were clearly visualized. Additionally, the aneurysmal neck, including the arteries of bifurcation, which were covered by the arch of the PulseRider for neck reconstruction, was clearly visualized. Figure 5 depicts a case where TOF-MRA failed to visualize the total site of the PulseRider placement; however, silent MRA was able to, excluding a segment of the proximal marker. Silent MRA at the 6-month follow-up clearly demonstrated occlusion of the residual neck; however, TOF-MRA follow-up images were similar to those of the initial session.

## Discussion

This study aimed to evaluate the visualization of coiled aneurysms with PulseRider, which is hindered due to metal artifacts in conventional TOF-MRA. Silent MRA successfully visualized intra-aneurysmal flow after PulseRider treatment, equivalent to that of DSA.

Metal artifacts are inevitable in assessing the occlusion status of cerebral aneurysms after endovascular surgery with coils and stents. PulseRider for wide-necked intractable cerebral aneurysms has been recently developed with high technical success [9]. Previous studies have assessed TOF-MRA images after PulseRider treatment; however, the visualization was poor due to metal artifacts [4, 5]. Although cerebral angiography is the gold standard for evaluating occlusion status after treatment, it is invasive, and there is a risk of thrombotic complications related to catheterization, contrast media, and exposure to radiation [10]. UTE-MRA has been introduced as a non-contrast MRA technique used to visualize flow signals superior to conventional TOF-MRA, even in flow-diverter stents with high levels of metal artifacts [6–8]. In our study, it was difficult to visualize the flow signal with TOF-MRA, especially at the neck where most of the PulseRider metal component exists. Although PulseRider contains less metal than a conventional stent [9], the unique design of PulseRider with concentrated metal arch coverage causes inevitable susceptibility artifacts at the aneurysm neck. The main metal component of the arch portion is nitinol, in addition to four radiopaque markers consisting of a platinum/iridium alloy. This metal concentration at the aneurysmal neck induces poor visualization of residual flow and the arteries of bifurcation. This is particularly an issue with the cavity around the neck of complex wide-necked aneurysms, which is important for detecting the risk of recanalization. In our study, silent MRA could visualize the flow signal of the remnant neck, as well as the arteries of bifurcation, which TOF-MRA failed to visualize. A segment of the proximal marker portion was poorly visualized even in silent MRA. Nguyen et al. reported that this is due to metal artifacts made of stainless steel at the detachment points [11]. Stainless steel causes more susceptibility artifacts than other metal components such as nitinol and platinum [12], and it hinders the evaluation of flow signal in segmental parent arteries even in silent MRA. However, other parts of the parent artery and the arteries of bifurcation, including the aneurysmal neck, were clearly visualized using silent MRA, equivalent to visualization using angiography. Meanwhile, the susceptibility artifact of stainless steel caused the risk of failure in evaluating the whole region of the PulseRider in TOF-MRA, as shown in one of the three basilar artery aneurysm cases in Fig. 5. We also assessed anterior communicating artery and middle cerebral artery aneurysms, which are more distal and smaller in size than the parent and branched arteries that cause more difficulty in visualization. Silent MRA can overcome this problem with satisfactory visualization of not only the residual cavity in the coiled aneurysms but also the parent and branched arteries.

Although the PulseRider has overall less metal than conventional nitinol neck-bridged stents; it has inevitable susceptibility artifacts due to its unique metal concentration at the aneurysm neck. Careful attention should be paid to focal loss of residual flow signal in TOF-MRA after PulseRider treatment, and silent MRA is a useful technique for avoiding the problem. This is the first report on the use of silent MRA for the evaluation of aneurysm occlusion status after PulseRider-assisted coil embolization, and we herein demonstrated its utility and feasibility. There are some limitations to this study. Only a few cerebral aneurysms were evaluated. All cases were treated with a T-shape PulseRider, and they were placed in extra-aneurysmal positions. Intra-aneurysmal placement may cause more metal artifacts.

In conclusion, non-contrast-enhanced UTE-MRA is a feasible imaging technique for cerebral aneurysms after PulseRider treatment. It can successfully visualize the residual cavity of coiled aneurysms treated with PulseRider better than TOF-MRA, and it may have the potential to provide visualization of aneurysm occlusion status equivalent to DSA.

## Data Availability

The data that support the findings of this study are available from the corresponding author upon reasonable request.
